# A Case of Diabetic Striatopathy Misdiagnosed as Intracranial Hemorrhage Showing the Importance of Early MRI

**DOI:** 10.1002/kjm2.70092

**Published:** 2025-08-17

**Authors:** Yi‐Ming Lu, I‐Hsaio Yang, Yoon Bin Chong

**Affiliations:** ^1^ College of Medicine, Kaohsiung Medical University Kaohsiung Taiwan; ^2^ Medical Imaging Department Kaohsiung Medical University Hospital Kaohsiung Taiwan; ^3^ Division of Neurosurgery, Department of Surgery Kaohsiung Medical University Hospital Kaohsiung Taiwan; ^4^ Graduate Institute of Medicine, College of Medicine, Kaohsiung Medical University Kaohsiung Taiwan

Dear Editor,

Although cases of diabetic striatopathy (DS) have been documented over several decades, it remains a rare diagnosis, with a reported prevalence of approximately 1 in 100,000. This rarity makes it an often underrecognized neurological complication of diabetes mellitus [1, 2]. Indeed, initial misdiagnosis of DS is common, with cases often being confused for acute stroke or intracranial hemorrhage [3]. Herein, we present the case of a patient with DS initially misdiagnosed as having an intracranial hemorrhage, highlighting the importance of early magnetic resonance imaging (MRI) and clinical awareness.

The patient was a 62‐year‐old male with poorly controlled diabetes mellitus, hypertension, chronic kidney disease stage 3, and a prior history of ischemic stroke who presented with acute onset of left‐sided involuntary movements, including frequent blinking, pronounced hand tremors, and irregular leg jerks. Neurological examination revealed prominent left‐sided dyskinesia and sensory deficits, consistent with diabetic neuropathy. Laboratory tests confirmed severe hyperglycemia (blood glucose: 384 mg/dL, HbA1c: 13.2%) and impaired renal function (creatinine: 2.04 mg/dL).

Initial computed tomography (CT) brain (Figure 1A) imaging revealed hyperdensity in the right lentiform nucleus, raising a suspicion of intracranial hemorrhage. However, subsequent MRI demonstrated characteristic imaging hallmarks of DS: a high signal intensity in the right lentiform nucleus on T1‐weighted imaging without contrast enhancement, mass effect, or edema (Figure 1B) and a high intensity signal in the right lentiform nucleus on T2‐weighted imaging (Figure 1C). This combination of findings—particularly the unilateral basal ganglia hyperintensity on T2‐weighted images without edema or enhancement—is crucial in differentiating DS from hemorrhagic or ischemic stroke, which often presents with mass effect, edema, or enhancement. Although the signal varies between patients, these features are common across patients with DS [1, 2, 4]. Early recognition and targeted glucose management allowed significant symptom improvement within a few days [5]. The patient's hemichorea markedly improved within days following strict glucose control and supportive care.

**FIGURE 1:**
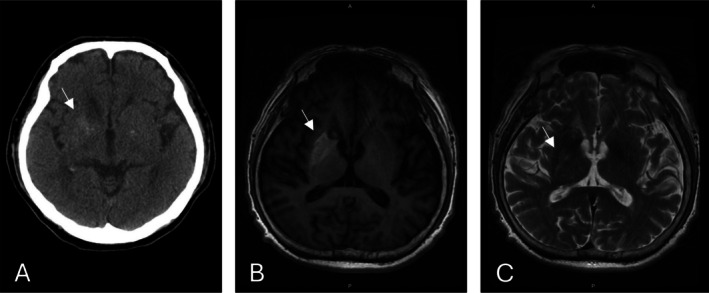
(A) Brain CT without contrast showing hyperdensity in the right lentiform nucleus (white arrow), (B) T1‐weighted fluid‐attenuated inversion recovery MRI of the brain showing a high signal intensity lesion in the right lentiform nucleus (white arrow), (C) T2‐weighted image showing a high intensity signal in the right lentiform nucleus (white arrow).

Overall, this case indicates that DS should be considered in diabetic patients presenting with acute choreiform movements. Key neuroimaging characteristics of DS include unilateral basal ganglia hyperdensity on CT scans without significant edema, and prominent hyperintensity on both T1‐weighted and T2‐weighted MRI scans, without enhancement or edema. In addition, the recognition of unilateral T2 hyperintensity in the basal ganglia, without any accompanying mass effect or enhancement, can provide valuable diagnostic clues, reinforcing the importance of MRI in the early and accurate identification of DS. Given the rarity and diagnostic challenges associated with this condition, a heightened awareness of DS is essential to avoid unnecessary invasive procedures and facilitate prompt, effective management.

## Conflicts of Interest

The authors declare no conflicts of interest.

## Data Availability

Data sharing is not applicable to this article as no new data were created or analyzed in this study.

## References

[1] A. Arecco , S. Ottaviani , M. Boschetti , P. Renzetti , and L. Marinelli , “Diabetic Striatopathy: An Updated Overview of Current Knowledge and Future Perspectives,” Journal of Endocrinological Investigation 47 (2024): 1–15. 10.1007/s40618-023-02166-5 PMC10776723 37578646

[2] C. B. Chua , C. K. Sun , C. W. Hsu , Y. C. Tai , C. Y. Liang , and I. T. Tsai , “‘Diabetic Striatopathy’: Clinical Presentations, Controversy, Pathogenesis, Treatments, and Outcomes,” Scientific Reports 10 (2020): 1594. 32005905 10.1038/s41598-020-58555-w PMC6994507

[3] I. Kwentoh , P. Win , F. Allayar , et al., “Diabetic Striatopathy: A Rare Case Report of Chorea, Hyperglycemia, Basal Ganglia (C‐H‐BG) Syndrome,” AIM Clinical Cases 3 (2024): 3.

[4] S. Dubey , P. Biswas , R. Ghosh , S. Chatterjee , B. Kanti Ray , and J. Benito‐León , “Neuroimaging of Diabetic Striatopathy: More Questions Than Answers,” European Neurology 85 (2022): 371–376. 35717942 10.1159/000524936

[5] S. H. Oh , K. Y. Lee , J. H. Im , and M. S. Lee , “Chorea Associated With Non‐Ketotic Hyperglycemia and Hyperintensity Basal Ganglia Lesion on T1‐Weighted Brain MRI Study: A Meta‐Analysis of 53 Cases Including Four Present Cases,” Journal of the Neurological Sciences 200 (2002): 57–62. 12127677 10.1016/s0022-510x(02)00133-8

